# Orthotopic model for the analysis of melanoma circulating tumor cells

**DOI:** 10.1038/s41598-024-58236-y

**Published:** 2024-04-03

**Authors:** Markéta Pícková, Zuzana Kahounová, Tomasz Radaszkiewicz, Jiřina Procházková, Radek Fedr, Michaela Nosková, Katarzyna Anna Radaszkiewicz, Petra Ovesná, Vítězslav Bryja, Karel Souček

**Affiliations:** 1 Department of Cytokinetics, Institute of Biophysics of the Czech Academy of Sciences, Brno, Czech Republic; 2 International Clinical Research Center, St. Anne’s University Hospital Brno, Brno, Czech Republic; 3 Department of Experimental Biology, Faculty of Science, Masaryk University, Brno, Czech Republic; 4 Institute of Biostatistics and Analyses, Faculty of Medicine, Masaryk University, Brno, Czech Republic; 5 Present Address: Centre for Inflammation Research, University of Edinburgh Institute for Regeneration and Repair, Edinburgh, Scotland

**Keywords:** In vivo model, Melanoma, Circulating tumor cells, Metastasis, Tumorectomy, Cancer, Cell biology, Melanoma

## Abstract

Metastatic melanoma, a highly lethal form of skin cancer, presents significant clinical challenges due to limited therapeutic options and high metastatic capacity. Recent studies have demonstrated that cancer dissemination can occur earlier, before the diagnosis of the primary tumor. The progress in understanding the kinetics of cancer dissemination is limited by the lack of animal models that accurately mimic disease progression. We have established a xenograft model of human melanoma that spontaneously metastasizes to lymph nodes and lungs. This model allows precise monitoring of melanoma progression and is suitable for the quantitative and qualitative analysis of circulating tumor cells (CTCs). We have validated a flow cytometry-based protocol for CTCs enumeration and isolation. We could demonstrate that (i) CTCs were detectable in the bloodstream from the fourth week after tumor initiation, coinciding with the lymph node metastases appearance, (ii) excision of the primary tumor accelerated the formation of metastases in lymph nodes and lungs as early as one-week post-surgery, accompanied by the increased numbers of CTCs, and (iii) CTCs change their surface protein signature. In summary, we present a model of human melanoma that can be effectively utilized for future drug efficacy studies.

## Introduction

Cutaneous melanoma is the most aggressive form of skin cancer and the 17th most commonly diagnosed cancer worldwide. It exhibits a high 5-year relative survival rate (98%) when diagnosed early in patients with localized disease. However, survival declines sharply in advanced melanoma patients with metastatic disease (5-year survival ~ 30%)^1,2^. Current treatment options for advanced melanoma include immune checkpoint blockade therapy and targeted molecular therapy^3^. However, the efficacy of these therapies is limited, highlighting the need for further research focused on identifying novel treatment strategies. To achieve this goal, we need to generate models of melanoma progression.

The suitable model should spontaneously metastasize to distant organs and allow analysis of the intermediate processes of metastasis formation, including the study of circulating tumor cells (CTCs). CTCs act as a critical mediator of cancer spread and, as such, can provide valuable information on disease progression and, vice versa, may inform about the sensitivity of cancer cells to tested drugs^4^. CTCs enumeration serves as a prognostic marker for metastatic disease and therapy resistance in breast, colorectal, and prostate cancer^5^. Similarly, the prognostic value of CTCs has been described in metastatic melanoma patients, where a high CTCs number correlates with an increased risk of disease progression, decreased overall survival, and reduced treatment response^6^. Kiniwa et al. conducted a clinical study on liquid biopsies from melanoma patients undergoing combined treatment with BRAF/MEK inhibitors, demonstrating the presence of CTCs even in the early stages of melanoma. Moreover, the number of CTCs reflects patients' responses to BRAF/MEK inhibitor treatment^7^. Recently, a specific microfluidic device for capturing melanoma CTCs was introduced^8^. However, both the understanding and the analysis of CTCs are limited due to challenges associated with the low abundance of CTCs, their phenotypic heterogeneity, the lack of reliable and reproducible detection and isolation techniques, and last but not least, the lack of suitable animal models for studies of melanoma-derived CTCs^9^.

Several mouse models mimicking metastatic melanoma have been introduced. One commonly used mouse model is the B16-F10 melanoma model. This highly aggressive and metastatic cell line was derived from a melanoma tumor in mice that metastasizes to distant organs such as the lungs, liver, and lymph nodes^10^. Another group of models, the genetically engineered mouse models of melanoma (GEMMs), carry the genetic changes seen in humans and show spontaneous metastasis, with behavior typical for the specific genetic modifications introduced^11^. However, the mouse origin of melanoma cells in these models represents an inherent limitation. Human melanoma is thus dominantly studied in xenografts, where human cell lines cultured in vitro are injected subcutaneously into immunodeficient mice^12^. The number of human xenograft melanoma models spontaneously developing metastases in mice is minimal^13,14^. The reliable mouse models that would allow simultaneous detection of melanoma dissemination and both qualitative and quantitative analysis of CTCs are entirely missing.

This study established a human orthotopic melanoma xenograft model using the metastatic A375 IV GFP Luc2 cell line, derived initially from lung metastatic foci in immunodeficient mice^15^. We comprehensively described the progression of melanoma from early to advanced metastatic stages in vivo and developed a flow cytometry-based protocol for CTCs detection and isolation from the whole blood. It opened the possibility of studying CTCs properties in detail and in parallel with monitoring both primary tumors and metastases. Our model of the metastatic process in melanoma is unique and especially suitable for the analysis of CTCs. We demonstrate that it can be used to analyze the dynamics and surface profile of human melanoma-derived CTCs at single-cell resolution.

## Material and methods

### Cells

A metastatic subline of the human A375 IV GFP cell line was cultured in Dulbecco’s modified Eagle’s medium (DMEM, 31966-021, Gibco, Life Technologies, Grand Island, NY) supplemented with 10% fetal bovine serum (FBS, 10270-106, Gibco, Life Technologies, Grand Island, NY) and 1% penicillin–streptomycin (XC-A4122/100, Biosera, Boussens, France) at 37 °C in a 5% CO_2_ atmosphere and 95% relative humidity. The cells were transfected with the pGL4.50 (luc2/CMV/Hygro) Vector (E1310, Promega, Madison, WI) containing resistance to Hygromycin B to enable stable luciferase expression and selection of clones in colony formation assays (Section "Colony formation assay"). Clonal lines were generated from single-cell-derived colonies and validated for GFP and firefly luciferase expression. The AmpFLSTR Identifiler PCR Amplification Kit (Applied Biosystems, ThermoFisher Scientific, Waltham, MA) was used to confirm the origin of cell lines. Cell lines were regularly tested for mycoplasma contamination using PCR.

### In vivo experiments

Male NOD.Cg-Rag1^tm1Mom^ Il2rg^tm1Wjl^/SzJ (NRG, The Jackson Laboratory, Bar Harbor, ME) mice aged six to eight weeks were anesthetized with a solution of ketamine (9.5 mg/ml; 99/192/85-C, Bioveta, Ivanovice na Hané, Czech Republic) and xylazine (0.95 mg/ml; A6A066, Bioveta, Ivanovice na Hané, Czech Republic) at a final dose of 100 µl/10 g of body weight, administered intraperitoneally. The mice were injected intradermally with 5 × 10^4^ A375 IV GFP Luc2 cells in 50 µl PBS. Live cell imaging was performed using the IVIS Lumina XR optical imaging system (Perkin Elmer, Waltham, MA) 15 min after intraperitoneal injection of D-luciferin, sodium salt (LUCNA-10G, Goldbio, St. Louis, MO, 150 mg/kg) with exposure times of 15 s, 30 s, and 60 s. Luminescence (total flux [p/s]) was quantified using Living Image v4.7.2 software (Perkin Elmer, Waltham, MA). All experiments were conducted with the approval of the Academy of Sciences of the Czech Republic (AVCR 103/2019), overseen by the ethical committee of the Institute of Biophysics CAS, performed by certified individuals (MP, RV), and carried out in accordance with relevant guidelines and regulations. All methods are reported in accordance with ARRIVE guidelines.

### Kinetic study

Five cohorts of mice were intradermally injected, as described above. Each week, one cohort consisting of n = 5 injected mice plus one intact mouse not injected with melanoma cells was euthanized. Whole blood was collected by cardiac puncture. Anesthetized mice were shaved in the chest area and disinfected with 70% ethanol. The 1 ml insulin syringe was flushed with 50 IU/ml heparin (4010809, Zentiva, Prague, Czech Republic) and injected into the right ventricle. The whole blood was collected into tubes containing 20 µl of 50 IU/ml heparin (4010809, Zentiva, Prague, Czech Republic). Flow cytometric analysis of CTCs was performed using a "no lyse/no wash" (NLNW) method (described in Section "Introduction of flow cytometric protocol for CTCs quantification from whole blood"), and a clonogenic assay was performed using blood samples and single-cell suspensions prepared from the lungs. GFP^+^ cells in the lungs were quantified using an Attune Classic flow cytometer (ThermoFisher Scientific, Waltham, MA).

### Tissue dissociation and analysis of DTCs

Lungs were mechanically minced using a gentleMACS™ Dissociator (Miltenyi Biotec, Bergisch Gladbach, Germany) and enzymatically digested with Collagenase Type I (2 mg/ml, CLS1 (LS004196), Worthington Biochemical Corporation, Lakewood, NJ) and Dispase II (0.75 mg/ml, 04942078001, Roche, Basel, Switzerland) at 37 °C for 1 h to obtain a single-cell suspension. The suspension was filtered through a 70 µm filter, washed with EDTA/PBS, and divided into two halves. One half was analyzed for GFP^+^ cells using flow cytometry, and the other half was used for colony formation assays.

Erythrocytes in the single-cell suspension were lysed with 1 × ACK buffer (1.5 M NH_4_Cl, 100 mM NaHCO_3_, 10 mM EDTA) and washed with PBS. Cells from the lungs were stained for viability using the LIVE/DEAD™ Fixable Aqua Dead Cell Stain Kit (L34957, Invitrogen™, ThermoFisher Scientific, Waltham, MA) for 20 min at 4 °C. After washing with PBS, viable GFP^+^ cells in the lungs were quantified using an Attune Classic flow cytometer (ThermoFisher Scientific, Waltham, MA).

### Flow cytometric detection of CTCs

Whole blood samples (200 µl) were stained with Hoechst 33342 (14533, Sigma-Aldrich, St. Louis, MO) at 10 µg/ml concentration for 15 min at room temperature, protected from light. Viability staining was performed using the LIVE/DEAD™ Fixable Red Dead Cell Stain Kit (Invitrogen, Carlsbad, CA) (Supplementary Table 1) for 20 min at 4 °C. The stained samples were diluted with 4 ml of PBS and measured using an Attune Classic flow cytometer equipped with 405 nm and 488 nm lasers at a flow rate of 500 µl/min (ThermoFisher Scientific, Waltham, MA). Erythrocytes were excluded from the analysis based on Hoechst 33342 and forward scatter (FSC) signals, allowing only nucleated cells to be recorded. Only viable single GFP^+^ cells without debris were included in the analysis. Data were analyzed using FlowJo software (Version 10.0.7, BD Biosciences, San Jose, CA).

### Spike experiment

A375 IV GFP Luc2 cells were spiked into 1 ml of whole blood obtained from intact animals at concentrations of 50, 500, 5000 and 50,000 cells. The samples were gently mixed by inversion, and 200 µl of each sample (i.e., 10, 100, 1000, 10,000 cells) was measured using an Attune Classic flow cytometer (ThermoFisher Scientific, Waltham, MA) or used for colony formation assay (Section "Colony formation assay").

### Colony formation assay

Blood collected by cardiac puncture was seeded into 10 ml of selection medium containing Hygromycin B (100 µg/ml, H3274, Sigma-Aldrich, St. Louis, MO). Similarly, half of the single-cell suspension prepared from the lungs was seeded in 10 ml of selection medium. Colonies were cultured for seven days at 37 °C, with the selection media changed twice a week. The grown colonies were fixed with methanol and stained with a 0.5% crystal violet solution in MQ water. Quantification was performed using automated image analysis in ImageJ software (NIH, Bethesda, MD, USA).

### Lung cryosections and immunofluorescence staining

To image micrometastases, dissected lung halves were fixed in 4% paraformaldehyde (PFA) in PBS, cryoprotected with 30% sucrose solution, and frozen in Tissue-Tek optimum cutting temperature (OCT) compound (25608-930, Sakura Fine-Tek, Torrance, CA). Ten-micrometer sections were used for immunofluorescence analysis. The frozen sections were permeabilized with 0.1% Triton X-100 in PBS, blocked with a 1% solution of bovine serum albumin in PBS, and then incubated overnight at 4 °C with primary antibody against GFP (20R-GR-011, Fitzgerald Industries International, Acton, MA) diluted in 1% BSA in PBS. Subsequently, the sections were incubated with a secondary antibody, Goat anti-Rabbit IgG (H + L) Highly Cross-Adsorbed Secondary Antibody, Alexa Fluor™ 488 (1:500, A-11034, Invitrogen, Carlsbad, CA), for 1 h at room temperature. Hoechst 33342 was used for nuclei staining (H1399, Thermo Fisher Scientific, Waltham, MA). After washing with PBS, the sections were mounted using a mounting medium (S3023, DAKO, Santa Clara, CA). Confocal laser scanning microscopy was performed using a Leica TCS SP8 platform (Leica Microsystems, Wetzlar, Germany).

### Flow cytometric characterization of melanoma heterogeneity

Primary tumors, lymph nodes, and lungs were enzymatically digested into single-cell suspensions to characterize melanoma heterogeneity as described above. Erythrocytes were lysed with 1 × ACK buffer (1.5 M NH_4_Cl, 100 mM NaHCO_3_, 10 mM EDTA) and washed with PBS. Cells from primary tumors, lymph nodes, lungs, and blood were stained for viability using the LIVE/DEAD™ Fixable Aqua Dead Cell Stain Kit (L34957, Invitrogen™, Carlsbad, CA) for 20 min at 4 °C. After washing with PBS, the cells were stained with a cocktail of primary antibodies or corresponding isotype controls, including EpCAM (2221100, SONY, Tokyo, Japan), Trop2 (1B-898-C100, Exbio, Vestec, Czech Republic), and CD271 (562122, BD Biosciences, San Jose, CA) (Supplementary Tables 1 and 2), as well as mouse IgG2b (400342, Biolegend, San Diego, CA), and mouse IgG1 κ (557872, BD Biosciences, San Jose, CA). After 20 min of incubation at 4 °C, staining with Streptavidin PE (12-4317, eBioscience, San Diego, CA) was performed for 20 min at 4 °C. The gating of positive populations was done using isotype controls. Only viable single GFP^+^ cells without debris were included in the analysis. The samples were measured on an Attune Classic flow cytometer (ThermoFisher Scientific, Waltham, MA) and analyzed using FlowJo software.

### Statistical analysis

Statistical analyses were performed in GraphPad Prism 9 software (GraphPad Software, Boston, MA) and R Statistical Software (v4.3.2; R Core Team 2023). Student's *t*-test was used to compare two experimental groups. ANOVA was used when comparing more than two groups. In the case of non-normal distribution, a non-parametric Kruskal's test and pairwise comparisons using Wilcoxon’s rank sum test with continuity correction have been used. A *p* value ≤ 0.05 was considered statistically significant.

## Results

### Intradermal injection leads to melanoma formation and dissemination within five weeks

**Figure 1:**
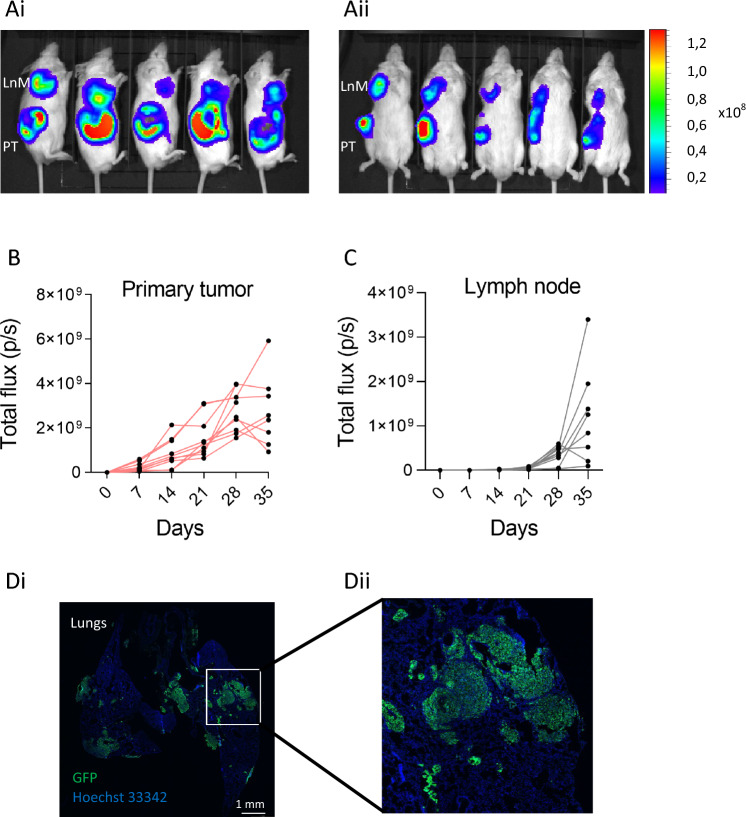
Characterization of A375 IV GFP Luc2—derived human melanoma xenograft. (**A**) Orthotopic melanoma xenografts and lymph node metastases were non-invasively continuously monitored in NRG mice. The representative images show a bioluminescent (BLI) signal in each mouse 35 days post-injection of melanoma cells from lateral (**Ai**) and ventral (**Aii**) view (PT ~ primary tumor, LnM ~ lymph node metastasis). (**B**, **C**) Quantification of bioluminescent signals from primary tumors (**B**) and lymph node metastases (**C**) in individual mice (n = 8). (**D**) Immunofluorescence of GFP in lungs (n = 12) dissected from animals 35 days post-injection. 10 × magnification (**Di**), detail of metastases (**Dii**). Images were taken using the confocal laser scanning microscopy platform Leica TCS SP8 (Leica), Tile Scan Acquisition Mode.

To establish a metastatic model of human melanoma, we utilized the A375 IV GFP cell line, known for its increased propensity to form lung metastases^15^. We generated the A375 IV GFP Luc2 cell line, which stably expresses the firefly luciferase, enabling the monitoring of melanoma progression in vivo. Intradermal injection of this cell line into one cohort of NRG mice and continuous monitoring within 35 days resulted in the development of solid primary tumors and lymph node metastases (Fig. 1A–C). The primary tumors exhibited continued growth from the injection day (Fig. 1B). Lymph node metastases became detectable through in vivo imaging at day 21 post-injection and displayed progressive growth until the study endpoint at day 35 (Fig. 1C). Histological analysis of lung cryosections collected at the endpoint (35 days post-injection) revealed the presence of metastatic lesions (Fig. 1D). In summary, we have successfully established a xenograft model of human melanoma characterized by continued progression and spontaneous metastases to lymph nodes and lungs.

### Introduction of flow cytometric protocol for CTCs quantification from whole blood

Having successfully established a model of melanoma progression with spontaneous metastases, our next objective was to develop a protocol for detecting and quantifying circulating tumor cells (CTCs), crucial mediators of tumor progression. Given the potential loss of CTCs during blood processing steps, we aimed to introduce a protocol for CTCs quantification directly from whole blood without the need for red blood cell lysis or additional washing and centrifugation steps.

**Figure 2:**
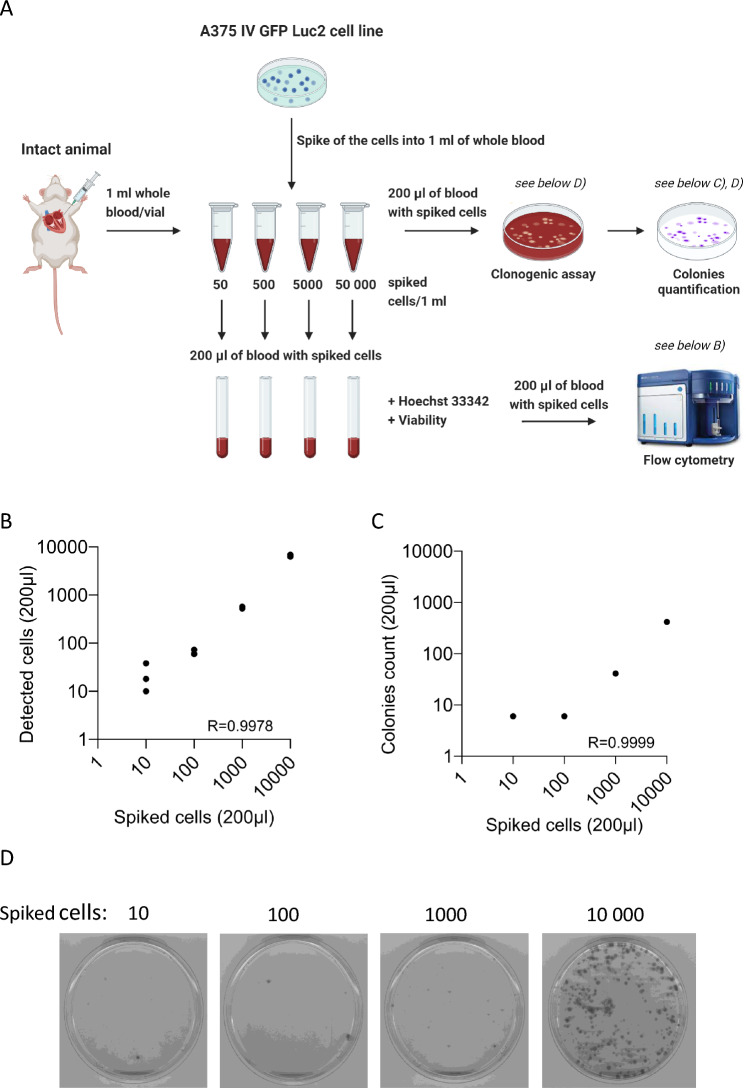
Validation of the method for quantification of CTCs from whole blood. (**A**) Experimental design of the optimization experiment for CTCs analysis from whole blood. A375 IV GFP Luc2 cells were spiked into whole blood. Created with BioRender.com. The spiked A375 IV GFP Luc2 cells were quantified using flow cytometry (**B**) and in vitro clonogenic assay (**C**, **D**). The flow cytometric detection of each concentration of spiked cells was done in technical triplicate. The grown colonies from an in vitro 1-week culture of spiked blood confirmed the presence of melanoma cells. (**C**) The colonies were stained with crystal violet and quantified using ImageJ software**.** The graphs show the correlation between the number of spiked cells and detected cells or grown colonies. (**D**) Example of images of grown colonies from in vitro culture.

To optimize the protocol, we spiked the A375 IV GFP Luc2 cell line into whole blood obtained from intact mice at concentrations ranging from 50 to 50,000 cells. A whole blood sample was stained with Hoechst 33342 to separate nucleated viable cells, and GFP-positive spiked CTCs were quantified using flow cytometry (Fig. 2A). As a reference method, we concurrently performed colony formation assays. The same sample was seeded into selection media, and after one week of cultivation, the resulting colonies were stained with crystal violet and quantified^16^.

To accurately identify the rare CTCs population from whole blood without the need for lysis and washing steps (referred to as no lyse-no wash, NLNW), a precise gating strategy was employed (Supplementary Fig. 1). In our experimental procedure, a crucial step involved the exclusion of erythrocytes from the acquired samples. To achieve this, we first visualized nucleated cells using Hoechst 33342 staining and then set an appropriate threshold on the detector for this marker to include only nucleated cells. Second, we employed a threshold on the cell size (forward scatter, FSC) detector. This combination of parameters allowed us to accurately distinguish erythrocytes from compact nucleated cells in whole blood samples. Third, a compact population of nuclear cells without doublets was gated. Fourth, viable GFP-positive cells were detected. Several positive and negative controls were prepared to establish the gating strategy: intact mouse blood served as the negative control. In contrast, blood spiked with dead cells was used to set the gate for viable/GFP-positive cells.

We then applied the established gating strategy to our set of spiked samples and observed a strong positive correlation between the number of spiked cells and the number of cells detected by our flow cytometry protocol (Fig. 2B). This data demonstrates our protocol's high sensitivity, allowing us to detect almost every spiked cell. Similarly, a positive correlation was observed between the number of CTCs quantified by flow cytometry in paired spike samples and the number of grown colonies (Fig. 2C,D). Data in Fig. 2C,D suggest that A375 IV GFP Luc2 cells retain high clonogenic capacity with tens of cells required for colony formation. In summary, these results confirm the suitability of our established flow cytometric protocol for the enumeration of CTCs from whole blood in our xenograft model of human melanoma.

### Dynamics of CTCs release during human melanoma xenograft progression

**Figure 3:**
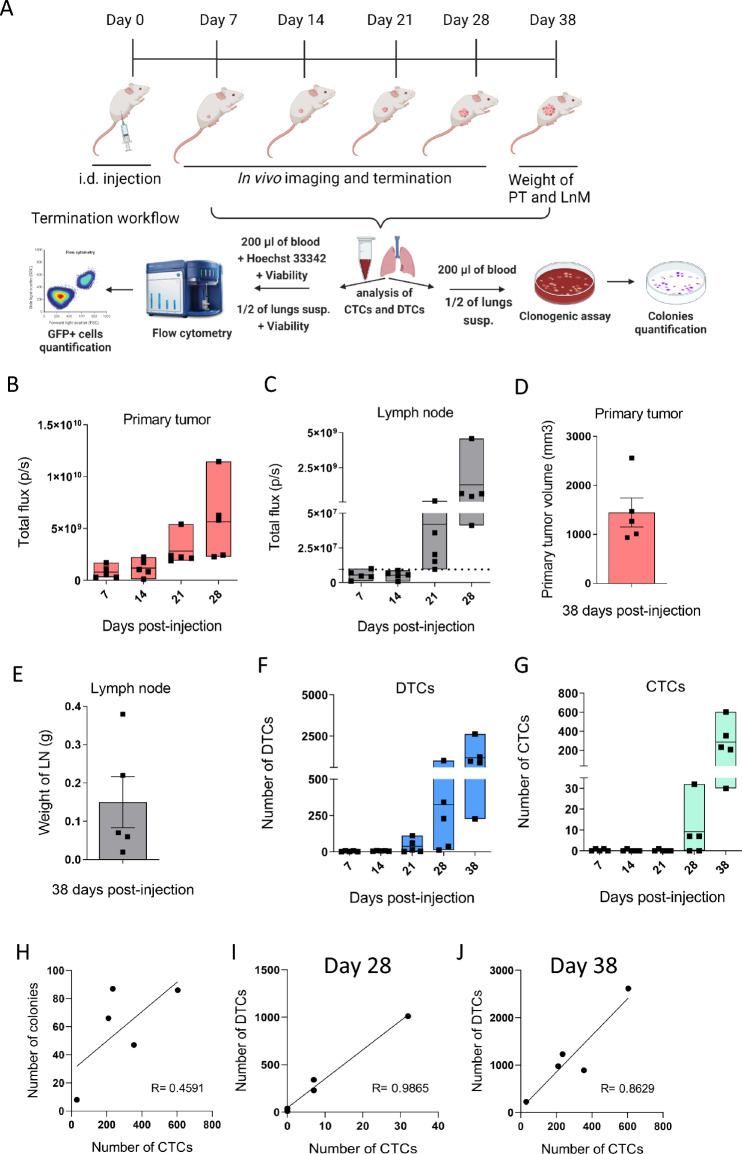
Dynamics of the melanoma progression from early to late stage. (**A**) Experimental workflow created with BioRender.com. (**B**, **C**) Orthotopic melanoma xenografts and the formation of lymph node metastases were non-invasively monitored in NRG mice. The graphs show quantified bioluminescent signals from primary tumor (**B**) and lymph node metastases (**C**) of individual animals from day 7 to day 28 post-injection of cancer cells (n = 5 per time point). The dotted line shows the mean bioluminescent signal of lymph nodes from a non-injected animal (intact) measured at each time point. (**D**) Primary tumor volumes were measured 38 days after injection. Mean ± SEM of primary tumor volume from individual animals (n = 5) is presented. (**E**) The weight of lymph nodes measured 38 days after injection. The graph shows mean ± SEM of lymph node weights from individual animals (n = 5). (**F**) Number of DTCs in lungs. The graph shows DTCs from individual animals from day 7 to day 38 post-injection of cancer cells (n = 5 per time point). (**G**) Number of CTCs detected in whole blood. CTCs counts from individual animals from day 7 to day 38 post-injection of cancer cells are presented (n = 5 per time point). Box ~ min–max, line ~ mean, dot ~ individual value. (**H**) Pearson´s correlation of the number of grown colonies in in vitro culture and the number of CTCs counted 38 days post-injection of cancer cells (paired analysis, n = 5). (**I**) Pearson´s correlation of the number of DTCs and the number of CTCs 28 days post-injection of cancer cells (paired analysis, n = 5). (**J**) Pearson´s correlation of the number of DTCs and number of CTCs 38 days post-injection of cancer cells (paired analysis, n = 5).

In the next step, we used an optimized CTCs quantification protocol to monitor the progression of human melanoma xenografts. First, we wanted to test a suitable site for blood collection for subsequent analysis of CTCs. The tail vein was suggested as the optimal site in terms of the potential for repeated collection from the same experimental animal. However, when testing different blood collection sites (tail vein, caudal artery, cardiac puncture) using the clonogenic assay, we found that the only reliable method for capturing CTCs is the well-established cardiac puncture (Supplementary Fig. 2A, B)^17^. Therefore, we injected five cohorts of mice as previously described, and each cohort was terminated at a different time point (day 7, 14, 21, 28, 38) (Fig. 3A).

Primary tumors exhibited continued growth from day 7 to day 28 after injection (Fig. 3B, Supplementary Fig. 2C,D), consistent with the dynamics observed in human melanoma xenograft progression monitored in a separate cohort of mice (Fig. 1). Detection of GFP signal by whole-body in vivo imaging is possible in this model but is much less robust and accurate than luciferase detection. It is due to the spectral characteristics of GFP and the proximity and autofluorescence of the tissue, limiting the penetration depth of fluorescence radiation and the lower efficiency of subsequent excitation. Despite these limitations, we monitored the GFP signal in vivo at timepoints 7, 14, 21, and 28 days after injection (Supplementary Fig. 2C–E). As expected, in vivo imaging by bioluminescence showed higher sensitivity compared to GFP. Therefore, we used luminescence detection for in vivo experiments and GFP detection for single-cell analysis using flow cytometry, where the limits mentioned above do not exist.

Lymph node metastases became detectable from day 21 after injection and their continued progression was monitored by in vivo imaging until day 28 (Fig. 3C, Supplementary Fig. 2C,E). Disseminated tumor cells (DTCs) were also detectable from day 21 post-injection, and they progressed to lung metastases at day 38 (Supplementary Fig. 3). Based on our experience, the maximum survival time for this xenograft model was 38 days. To model the very late stage of melanoma, we terminated the last cohort at this time point. However, primary tumors at this advanced stage exhibited necrosis, which affected the quality of bioluminescence signal detection. Therefore, to obtain more accurate information about tumor growth, we measured the volume of primary tumors (Fig. 3D) and weighed the lymph nodes (Fig. 3E). CTCs in blood and lung DTCs were analyzed using the same methods as in previous time points. The burden of DTCs in the lungs consistently increased up to day 38 after injection (Fig. 3F). Interestingly, CTCs were detectable in three animals from day 28 after injection, while all animals exhibited high levels of CTCs in circulation 38 days after injection (Fig. 3G, H). Moreover, we observed a positive correlation between the number of DTCs and CTCs at the advanced stage of the metastatic melanoma xenograft model starting 28 days post-injection (Fig. 3I, J).

These results suggest that primary tumors and metastases in the lymph nodes and lungs progress from the early stages of the disease. At the same time, detectable numbers of CTCs are associated with advanced melanoma stages.

### Dissemination of melanoma cells and the impact of primary tumor resection on CTCs and metastatic progression

The cancer dissemination and subsequent metastatic outgrowth have long been considered events associated with advanced stages of cancer. However, in the last decade, it has been established that the dissemination of cancer cells occurs even in the early stages, preceding the diagnosis of the primary tumor^18,19^.

**Figure 4:**
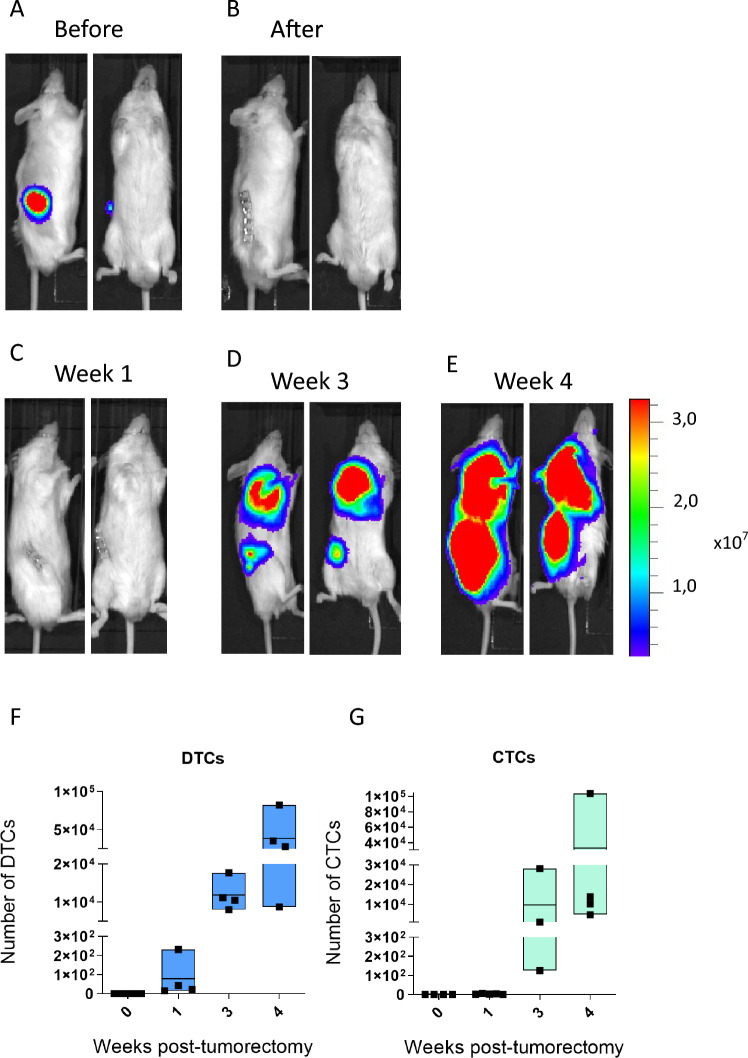
Melanoma progression after tumorectomy. (**A**–**E**) The melanoma xenograft progression was non-invasively monitored in NRG mice after resection of primary tumors. Representative images show a bioluminescent signal before (**A**) and after (**B**) surgery. The exposure time for in vivo imaging was 15 s. The disease progression is shown as one (**C**), three (**D**), and four (**E**) weeks post-tumorectomy, *n* = 4 mice per group. (**F**) Quantification of DTCs in lungs in individual animals from week one after tumorectomy to week four (n = 4 per time point). (**G**) Quantification of CTCs from whole blood of individual animals from week one to week four post-tumorectomy (n = 4 per time point). Box ~ min–max, line ~ mean, dot ~ individual value.

To investigate whether the dissemination of CTCs occurs early in melanoma progression in our model, we chose to surgically resect the primary tumor (PT) and monitor the growth of metastases. The earliest time point for surgical resection was 14 days after injection of melanoma cells when the primary tumors were solid and localized (Fig. 4A,B). Subsequently, to simulate the clinical scenario, we monitored the metastatic progression after tumor resection until the animals exhibited signs of cachexia (Fig. 4C–E). In vivo imaging showed rapid progression of lymph node metastases within three weeks (Fig. 4D) and lung metastases within four weeks (Fig. 4E) after tumor resection. However, single-cell flow cytometric analysis of DTCs in the lung showed tumor cells as early as one week after resection (Fig. 4F).

Interestingly, we did not detect any CTCs in the blood at the early time point (Fig. 4G), but three and four weeks after PT resection, CTCs numbers increased and correlated with continuously growing numbers of DTCs (Fig. 4F,G). Comparison of corresponding time intervals without (3 and 5 weeks) and with (2 + 1 and 2 + 3 weeks) PT resection associates with an increased number of DTCs, which was significant after five weeks (Supplementary Fig. 4A). The number of CTCs followed this trend but was not statistically significant (Supplementary Fig. 4B).

In summary, results demonstrate that melanoma cells in this model disseminate very early, although CTCs may remain below the assay's detection limit. The surgical resection of primary tumors in other malignancies can lead to metastatic outgrowth and disease progression^20,21^. We simulated a similar situation in the presented model and observed a substantial increase in detected DTCs and elevation in CTCs three weeks after tumorectomy.

### Surface profile characterization of melanoma cells in the metastatic cascade

**Figure 5:**
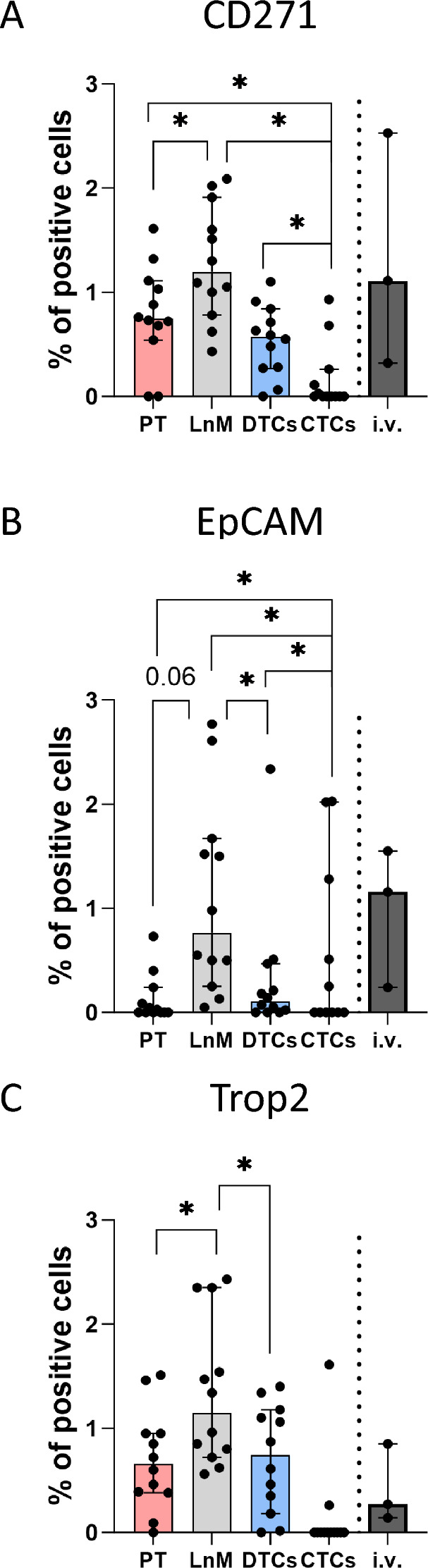
Single-cell characterization of A375 IV GFP Luc2 at day 38 post injection. Analysis of surface markers related to melanoma progression and EMT on melanoma cells from primary tumors (PT), lymph node metastases (LnM), disseminated tumor cells (DTCs), circulating tumor cells (CTCs), and in vitro cultivated A375 IV GFP Luc2 cells (i.v.). Characterization of expression of CD271 (**A**), EpCaM (**B**), and Trop2 (**C**) on melanoma cells. Tissue and blood samples were harvested from the animals 38 days after i.d. injection of the cells (two independent repetitions, total n = 17, i.v. ~ n = 3), processed and stained as described in the Material and Methods section, and analyzed using flow cytometry. Viable, single GFP^+^ cells without debris were taken into analysis. The graphs show medians ± 95% CI of % of positive cells, staining for individual animals. Non-parametric Kruskal test and Pairwise comparisons using Wilcoxon rank sum test with continuity correction have been used for statistical analysis (i.v. group not included), *p < 0.05.

Cancer heterogeneity poses a significant challenge in cancer treatment and CTCs detection. Specific changes in the expression of surface markers, particularly on CTCs, serve as prognostic indicators of disease status and response to therapy^9,22^. Therefore, our next goal was to demonstrate that an established protocol for detecting and quantifying CTCs by GFP-based flow cytometry can be extended to detect the expression of selected surface molecules characterizing their phenotype. At the same time, we wanted to compare the surface expression profile of melanoma cells in individual tissues, i.e., primary tumor (PT), lymph node metastasis (LnM), tumor cells disseminated to the lungs (DTCs), and circulating in the blood (CTCs). For the analysis, we selected features specific to melanoma—CD271^23^ and features associated with the phenotypic plasticity—EpCAM^24^ and its homolog Trop2^25^, which are known to be related to the epithelial phenotype regulated by the epithelial-to-mesenchymal transition/ mesenchymal-to-epithelial transition (EMT/MET)^26^. The multi-color protocol was successfully introduced and validated using the A375 cell line cultured under in vitro conditions (Supplementary Table 1). Subsequently, flow cytometric analysis of individual GFP^+^ cells in samples was performed after dissociation of the above tissues 38 days after intradermal injection of A375 GFP Luc2 cells. CD271 expression was comparable on the surface of cells from PT, LnM, DTCs, and CTCs (Fig. 5A). As a quantifiable parameter, we chose the % of positive cells, which provided the most robust output despite their relatively low frequency. The expression of all surface molecules showed considerable plasticity during the dissemination process. The fraction of CD271^+^ cells was significantly reduced in CTCs compared with PT, LnM, and DTCs cells (Fig. 5A). Interestingly, EpCAM^+^ cells were explicitly increased in LnM and CTCs compared to PTs (Fig. 5B). The frequency of Trop2^+^ cells was increased in LnM compared with PT and DTCs; the decrease in CTCs is visible but statistically not proved to be significant (Fig. 5C).

In summary, our protocol enables the detection of specific surface markers. Additionally, we have demonstrated that melanoma cell phenotype is plastic during progression from primary tumor to distant metastases.

## Discussion

Metastatic melanoma is a highly aggressive form of skin cancer, and the development of comprehensive animal models that accurately mimic disease progression is crucial for understanding its dynamics and identifying effective therapeutic strategies^12,27^. In this study, we successfully established a spontaneously metastatic xenograft model of human melanoma that recapitulates disease progression and can be utilized for future drug efficacy studies. Additionally, we provide evidence that the newly developed model is especially suitable for the analysis of CTCs. Using a flow cytometry-based protocol for detecting and quantifying CTCs in melanoma, we could characterize CTCs at different stages of the metastatic cascade. We provide evidence that our model overcomes the limitations of traditional subcutaneous injection models by better recapitulating the progression steps and disease dynamics seen in patients.

Additionally, it can be utilized for various preclinical studies focusing on non-immune-based therapies and BRAF-targeted therapies, which are commonly applied to melanoma patients. Still, resistance to these treatments is often acquired by melanoma cells^28,29^. Despite all these benefits for CTCs-melanoma studies, we must acknowledge the challenges of translating our model and methodology into the clinic. The immune system plays a dual role in cancer progression because it suppresses tumor spread through immune surveillance and paradoxically facilitates the spread of tumor cells through circulation^30^. At the same time, CTCs express immune checkpoint regulators and markers that can aid them in evading immune surveillance or modulating immune response^31^. Therefore, we are aware of the limitation through the deficiency of T, B, and NK cells in the mouse strain we utilized in this study^32^. From this perspective, it is essential to note that even humanized mouse models have limitations in cancer research. These include incomplete reconstruction of the human immune system, under-representation of the human tumor microenvironment, and species-specific differences in genetics and physiology^33^. This model allows engraftment of human cells and provides an accessible in vivo setup to observe drug sensitivity of melanoma tumor cells.

CTCs are critical in cancer dissemination and can serve as prognostic markers for metastatic disease. However, only little is known about the biology of CTCs, and some key questions are still being debated^34^. For example, what are the mechanisms controlling the release and maintenance of CTCs? Is there a unique molecular CTC signature? Our model, combined with a flow cytometry-based protocol for quantifying CTCs directly from whole blood, provides the tool and capacity to answer some of these burning questions. Although the intracardiac blood collection method can be used for animal studies, it mimics human patient blood collected from the cubital vein. Circulating blood is used to analyze CTCs in both cases. The reliable detection and isolation of melanoma CTCs in our model allows us to overcome the challenges associated with the low abundance and phenotypic heterogeneity of CTCs. We could monitor the progression, metastases formation, and CTCs release in parallel over time.

The LN metastases and CTCs in the bloodstream appeared at the same time as early as four weeks after engraftment. This finding is consistent with kinetics studies on breast cancer models, where CTCs were detectable when macrometastases appeared^35,36^. Additionally, we observed a strong correlation between the number of CTCs and DTCs in the lungs. The appearance of CTCs only in later stages, when macrometastases developed, suggests that metastatic foci serve as a new source of CTCs^4,37^ and supports the accepted notion that CTCs are prognostic indicators for metastatic melanoma patients^38,39^.

In most solid tumors, cancer cells disseminate via the lymphatic system before metastasizing through the vascular system^40,41^. This has been confirmed in an animal model of breast cancer metastasis. It was observed that tumor cells that metastasize through the lymphatic system differ from those that spread through the bloodstream. This could potentially explain why surgical resection of the primary tumor can result in different dynamics of accelerated metastatic progression and increased release of CTCs. It also suggests a potential role for surgical intervention in promoting the spread of cancer cells^42^. The rapid progression towards metastases and a significant increase in CTCs following tumor resection supports the hypothesis that metastatic lesions function as a source of aggressive CTCs in our model, detectable in the terminal stages of the disease^42^.

A375 lines isolated from malignant melanoma are poorly metastatic in vivo and produce few lung metastases when injected into immunodeficient mice circulation^13^. To establish an experimental metastatic model of human melanoma, we used a highly metastatic subline known to form lung metastases efficiently^15^. A limitation of this model may be that we are using an already preselected and highly malignant population on the primary tumor side. Thus, this model does not recapitulate the view when metastases arise from rare populations within the primary tumor. Rather, it corresponds to the situation where the primary tumor shows a phenotype with a propensity to metastasize, and the ability to metastasize is determined early in primary tumor development and does not require further selections among primary tumor cells^13,43^. The essential advantage of our experimental setup is the capacity to isolate and characterize individual melanoma CTCs reliably. We could describe critical features of melanoma cells at the single-cell level at different stages of the metastatic cascade. Immunophenotyping has provided valuable insights into the heterogeneity of melanoma cells and their potential therapeutic vulnerabilities^44^. We have focused our attention on the epithelial markers EpCAM and its homolog Trop2 and the marker of highly progressive melanoma stem cells, CD271^45,46^. All analyzed populations defined based on the positive expression of selected molecules showed considerable dynamics during dissemination. In particular, the dynamics of Trop2^+^ population size and the observed variability in the frequency of EpCAM^+^ CTCs have been observed in other experimental models and confirm the limitations of CTCs detection methods based only on the detection of selected surface molecules (e.g., EpCAM)^47^.

These findings demonstrate the compatibility of our protocol with surface marker staining and, importantly, suggest the plasticity of cancer cells during their progression from the primary tumor to metastases.

In conclusion, we have successfully characterized an orthotopic xenograft model of human melanoma that accurately recapitulates critical steps of disease progression, spontaneously metastasizes to lungs and lymph nodes and allows parallel and sensitive detection of CTCs. Melanoma’s CTCs detection, quantification, and isolation directly from whole blood represent a unique feature of the model and provide an efficient tool for studies focused on biology and targeting of CTCs. We are convinced that our model will contribute to a better understanding of melanoma biology and will guide the development of targeted therapies focused on melanoma progression that will infinitely improve the prognosis of patients with this deadly disease.

### Supplementary Information

Supplementary Legends.

Supplementary Figures.

Supplementary Tables.

## Data Availability

The datasets generated during this study are available from the corresponding author upon reasonable request.
