# Supplementary material for: Orthotopic model for the analysis of melanoma circulating tumor cells
Source: Sci Rep. 2024 Apr 3;14:7827. doi: 10.1038/s41598-024-58236-y (PMC10991390; doi:10.1038/s41598-024-58236-y)
Supplement: Supplementary file 1 — Supplementary Legends. [file 41598_2024_58236_MOESM1_ESM.docx]

**Picková, M. et al., Supplementary Figure Legends**

**Supplementary Figure 1:** **Gating strategy of CTCs population detected by flow cytometry.** The gating strategy was set using negative (Intact blood) and positive (Spiked blood) controls. Dead cells were spiked in whole blood for viability testing. The population of GFP^+^ cells (CTCs) was gated from the compact population of single-nucleated cells.

**Supplementary Figure 2: Dynamics of the melanoma progression from early to late stage. (A)** Experimental design of blood collection by cardiac puncture (i.c.), tail vein (t.v.), and caudal artery (ca.a.) 35 days post-injection of melanoma cells. Created with BioRender.com. **(B)** Images of grown colonies from *in vitro* culture stained with crystal violet. **(C)** Orthotopic melanoma xenografts and lymph node metastases were non-invasively monitored from day 7 to day 28 post-injection of cancer cells using both bioluminescent signal (upper panel) and GFP fluorescent signal (lower panel) from primary tumors and lymph node metastasis (n=5 per time point; in the image are four injected mice + one intact mouse). **(D, E)** Quantification of GFP fluorescent signal from primary tumors **(D)** and lymph nodes, respectively **(E),** from day 7 to day 28 post-injection of melanoma cells (n = 5). The data are presented as total flux (photons/second) from individual animals. Box ~ min-max, line ~ mean, dot ~ individual value.

**Supplementary Figure 3: Dynamics of the melanoma progression from early to late stage.** DTCs were detectable by flow cytometry and clonogenic assay from day 21 post-injection and progressed in metastases until day 38. **(A)** The population of live single DTCs in each time point for individual mice. As a control, a non-injected animal (intact) was used. **(B)** Representative images of grown colonies from *in vitro* cultured DTCs stained with crystal violet.

**Supplementary Figure 4:** **Melanoma progression after tumorectomy**

Numbers of DTCs without (W/O) and post-tumorectomy were compared in two-time points. **(A)** Number of DTCs in lungs. **(B)** Numbers of CTCs in whole blood. The graph shows CTCs or DTCs from individual animals at week 3 (2+1 weeks post-tumorectomy) and at week 5 (2+3 weeks post-tumorectomy) n = 3-4 per time point, *~ p<0.05. The data used are the same as in Figure 4. Box ~ min-max, line ~ mean, dot ~ individual value.

**Supplementary Table 1:** Overview of antibodies and reagents used for flow cytometry experiments.

**Supplementary Table 2:** MIFlowCyt-Compliant Items.
