# Supplementary material for: Orthotopic model for the analysis of melanoma circulating tumor cells
Source: Sci Rep. 2024 Apr 3;14:7827. doi: 10.1038/s41598-024-58236-y (PMC10991390; doi:10.1038/s41598-024-58236-y)
Supplement: Supplementary file 2 — Supplementary Figures. [file 41598_2024_58236_MOESM2_ESM.pdf]

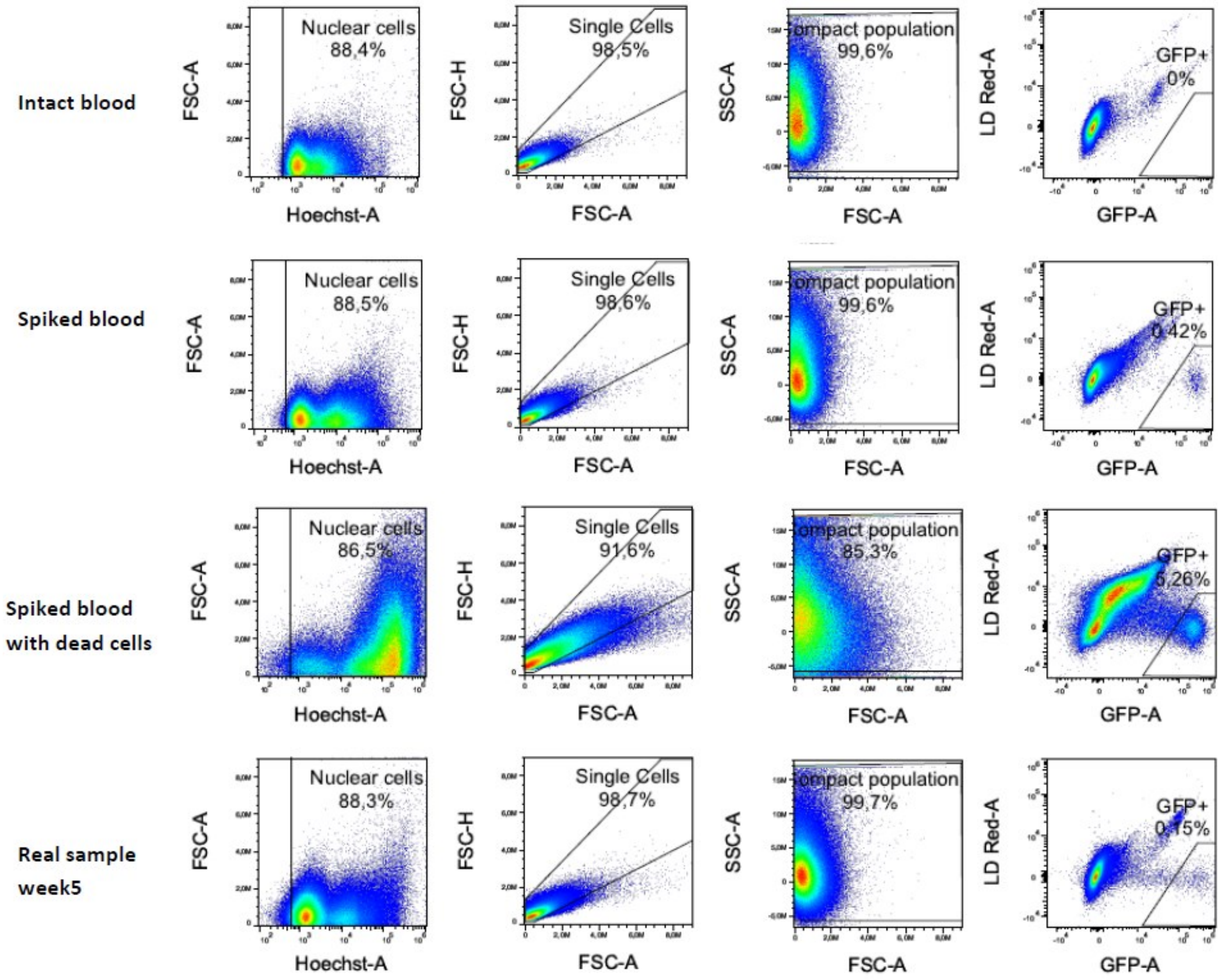

Supplementary Figure 1

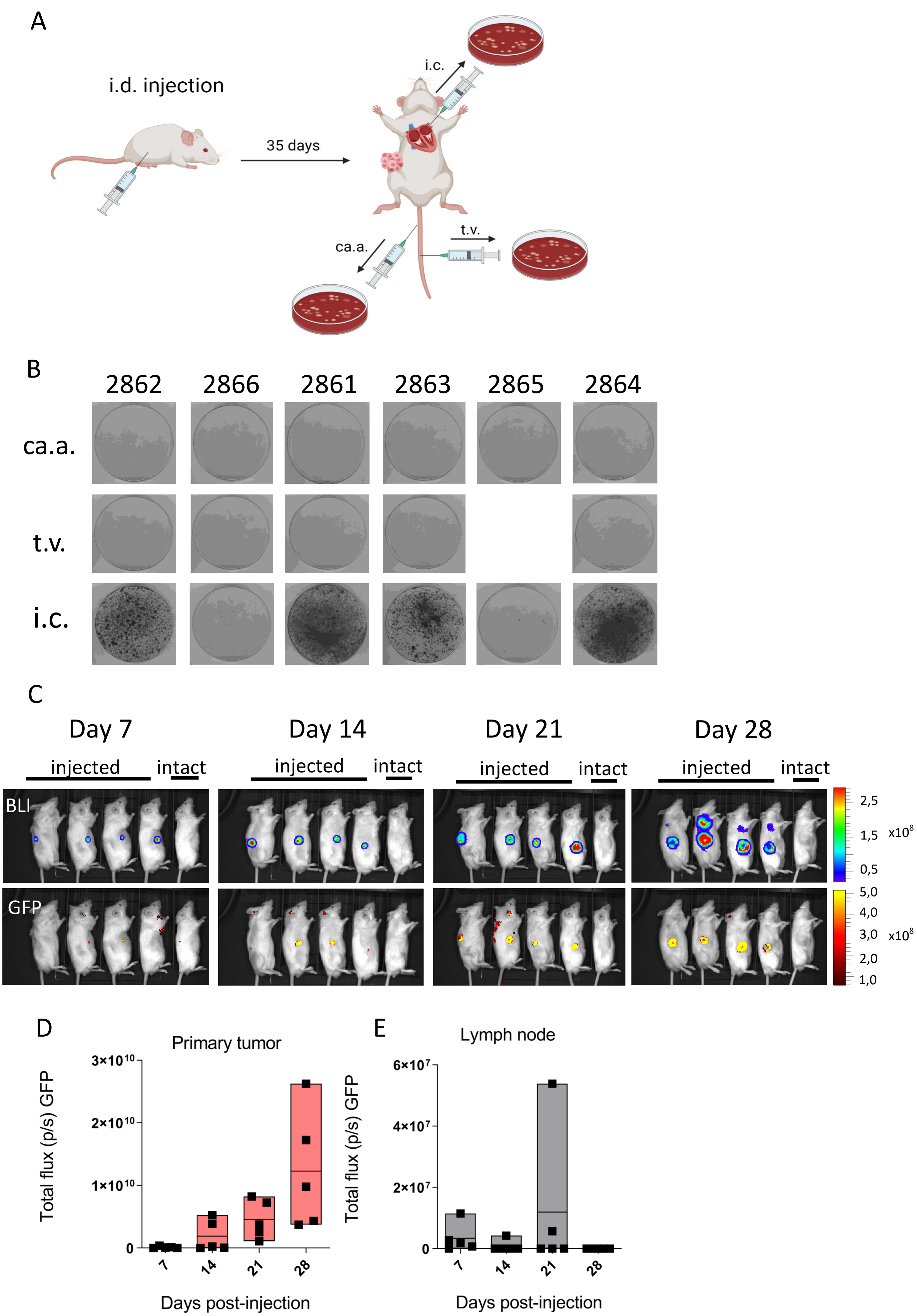

Supplementary Figure 2

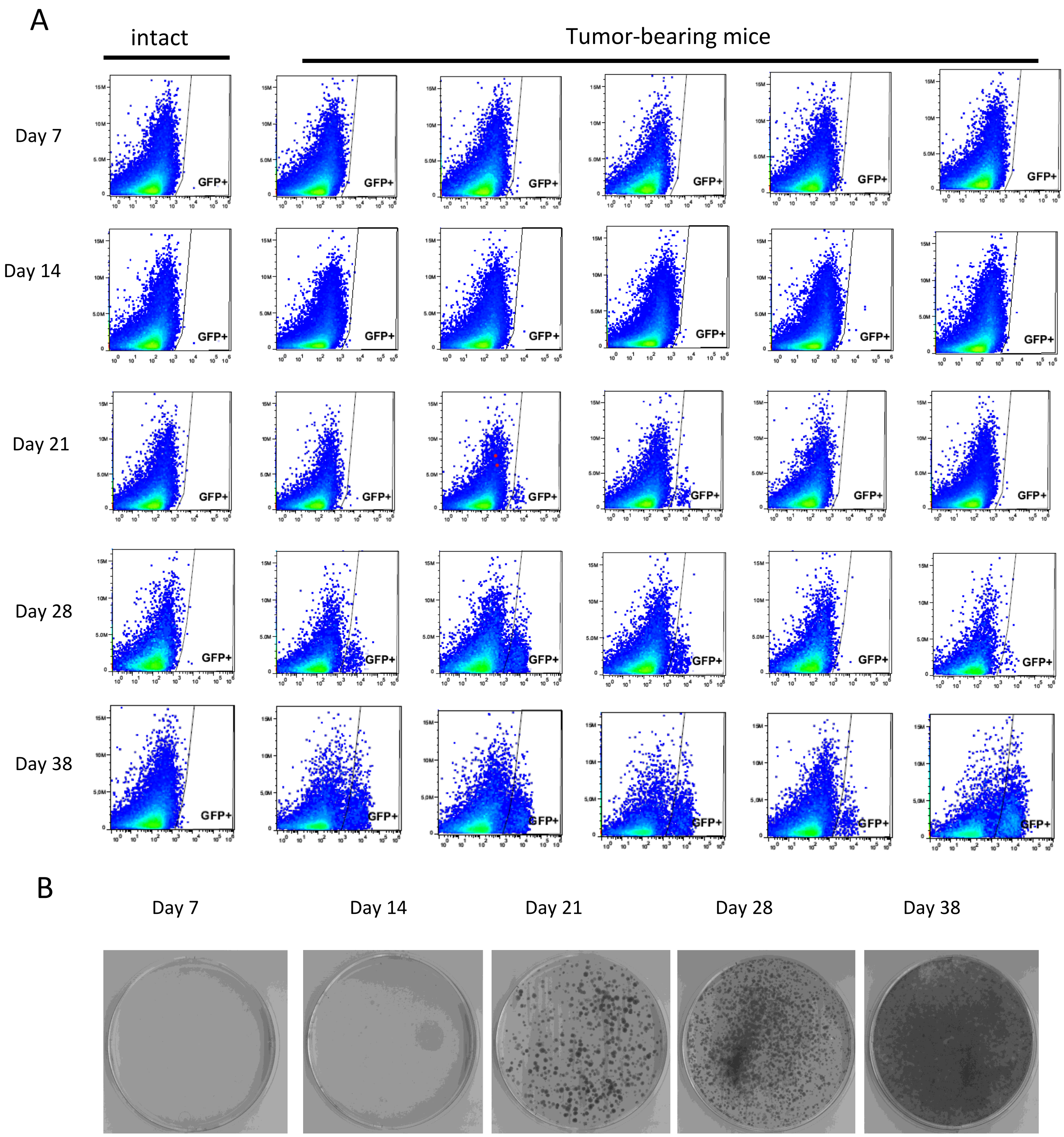

Supplementary Figure 3

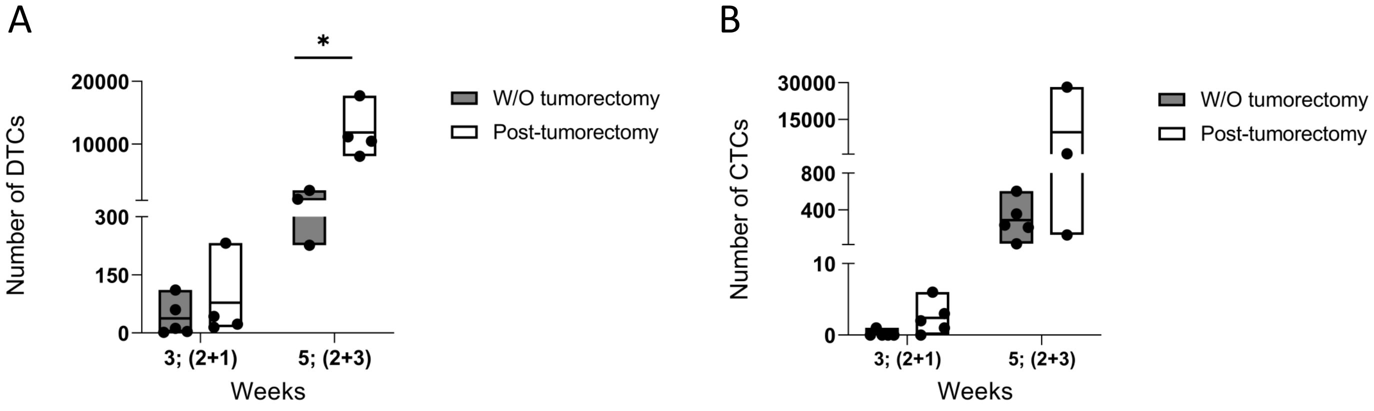

Supplementary Figure 4
