# Supplementary material for: Orthotopic model for the analysis of melanoma circulating tumor cells
Source: Sci Rep. 2024 Apr 3;14:7827. doi: 10.1038/s41598-024-58236-y (PMC10991390; doi:10.1038/s41598-024-58236-y)
Supplement: Supplementary file 3 — Supplementary Tables. [file 41598_2024_58236_MOESM3_ESM.pdf]

Supplementary Table 1: Overview of antibodies and reagents used for flow cytometry

marker	conjugate	source	company	cat. number	concentration	dilution
**primary antibodies**						
EpCAM	BV421	Mouse	SONY	2221100	50 µg/ml	1:20
CD271	PE-Cy7	Mouse	BD Biosciences	562122	-	1:100
Trop2	biotin	Mouse	Exbio	1B-898-C100	1 mg/ml	1:250
**isotype controls**						
Mouse IgG2b	BV421	Mouse	Biolegend	400342	50 µg/ml	1:20
Mouse IgG1 κ	PE-Cy7	Mouse	BD Biosciences	557872	-	1:100
**secondary detection**						
streptavidin	PE	-	eBioscience	12-4317	0.2 mg/ml	1:2000
**viability**						
LIVE/DEAD Fixable Red Dead Cell Stain		-	ThermoFisher Scientific	L23102	-	1:500
LIVE/DEAD Fixable Aqua Dead Cell Stain		-	ThermoFisher Scientific	L34957	-	1:500
**DNA stain**						
Hoechst 33342	-		Sigma-Aldrich	14533	5 mg/ml	1:500

Author Checklist: MIFlowCyt-Compliant Items

Requirement	Please Include Requested Information
1.1. Purpose	The purpose of this study was to introduce flow cytometric “no lyse no wash” protocol for detection and quantification of circulating tumor cells (CTCs) in whole blood of mouse xenograft model of melanoma. We combined detection of GFP reporter protein in A375 IV luc GFP cells, nucleated cells (labelled with Hoechst 33342) and viability (fixable viability probe).
1.2. Keywords	in vivo model, melanoma, circulating tumor cells, metastasis, tumorectomy
1.3. Experiment variables	A375 IV luc GFP human melanoma cells derived from lung metastases of immunodeficient mouse and A375 luc human melanoma cells were used for establishment and optimization of particular steps of “no lyse no wash” protocol in this study.
1.4. Organization name and address	Department of Cytokinetics, Institute of Biophysics of the CAS, v.v.i., Královopolská 135, 612 65 Brno, Czech Republic<br><br>International Clinical Research Center, St. Anne’s University Hospital Brno, Pekařská 53, 656 91 Brno, Czech Republic<br><br>Department of Experimental Biology, Faculty of Science, Masaryk University, Kotlářská 2, 611 37 Brno, Czech Republic
1.5. Primary contact name and email address	Karel Souček, Ph.D.<br><br>ksoucek@ibp.cz
1.6. Date or time period of experiment	1.1.2020 – 31.12.2022
1.7. Conclusions	We have established a flow cytometric “no lyse no wash” protocol for detection and quantification of circulating tumor cells (CTCs) in whole blood of mouse xenograft model of melanoma. Further we characterized selected surface markers on melanoma cells in metastatic cascade.
1.8. Quality control measures	Performance and stability of Attune classic flow cytometer was assured by performing Performance Quality Control before 1st measurement of the day using Attune Performance Tracking beads (#4449754), as recommended by manufacturer.
2.1.1.1. (2.1.2.1., 2.1.3.1.) Sample description	Human adherent melanoma cell line A375 IV luc GFP and A375 luc were propagated in *in vitro* conditions. A375 IV luc GFP was intradermally injected into immunodeficient animals to generate primary tumors and CTCs.
2.1.1.2. Biological	A375 IV GFP cell line was a gift from Dr. L. Kučerová, Laboratory of Molecular Oncology, Cancer Research Institute, Slovak Academy of Sciences, Bratislava, Slovakia

<table>
<tr><td>sample source description</td><td></td></tr>
<tr><td>2.1.1.3. Biological sample source organism description</td><td>A375 cell line was established from a 54-year-old female with malignant melanoma. Its metastatic derivate A375 IV GFP was generated by in vivo propagation of the parental cell line in immunodeficient mice and isolated from metastatic lesions in lungs. A375 luc and A375 IV luc GFP were generated by transfection of cells with the pGL4.50 (luc2/CMV/Hygro) Vector (E1310, Promega).</td></tr>
<tr><td>2.1.2.2. Environmental sample location</td><td>-</td></tr>
<tr><td>2.3. Sample treatment description</td><td>There was no treatment of the cells used in this study.</td></tr>
<tr><td>2.4. Fluorescence reagent(s) description</td><td>For flow cytometric analysis, single cell suspension of cell line was obtained using EDTA/PBS and Trypsin/EDTA incubation. Cells spiked into intact whole blood (controls) or whole blood with CTCs from tumor bearing animals were stained with following reagents:

- LIVE/DEAD Fixable Red Dead Cell Stain Kit (L23102, ThermoFisher Scientific), Hoechst 33342 (14533, Sigma-Aldrich)

For flow cytometric characterization of cells from tissue, were prepared single cell suspension after enzymatic digestion with collagenase type I and dispase. Single Cell suspensions were prepared and stained with following antibodies and viability:
- Dispase II (Roche, 04942078001), Collagenase type I (Worthington, LS004176), DNAse I (Roche, 10104159001), LIVE/DEAD Fixable Aqua Dead Cell Stain Kit (L34957, ThermoFisher Scientific), EpCAM (SONY, 2221100), CD271 (BD Bioscience, 562122), Trop2 (Exbio, 1B-898-C100), Mouse IgG2b (Biolegend, 400342), Mouse IgG1 κ (BD bioscience, 557872), Strepatavidin-PE (12-4317, eBioscience)</td></tr>
<tr><td>3.1. Instrument manufacturer</td><td>Attune® Acoustic Focusing Cytometer – Life Technologies Corporation</td></tr>
<tr><td>3.2. Instrument model</td><td>Attune® Acoustic Focusing Cytometer, installation date: 2012</td></tr>
<tr><td>3.3. Instrument configuration and settings</td><td><table><tr><th>Marker</th><th>Fluorochrome</th><th>Source</th><th>Company</th><th>Catalog Number</th><th>Antibody concentration</th><th>Dilution</th><th>Laser</th><th>Excitation maxima (nm)</th><th>Emission maxima (nm)</th><th>Manufacturer</th><th>Power</th><th>Instalation date</th><th>Detector</th><th>Dichroic Mirror // Band Pass</th><th>Instrument</th></tr></table></td></tr>
</table>

														Filter (nm)	
EpCAM	BV421	Mouse	SONY	2221100	Lot specific	1:20	405	405	421	Life Technologies Corporation	50mW	2012	VL1	410 DLP // 450/40	Attune® Acoustic Focusing Cytometer
CD271	PE-Cy7	Mouse	BD bioscience	562122	-	1:100	488	561	780	Life Technologies Corporation	20mW	2012	BL3	620 DLP // 640LP	Attune® Acoustic Focusing Cytometer
Trop2	Biotin	Mouse	Exbio	1B-898-C100	1 mg/ml	1:250	-			Life Technologies Corporation	20mW	2012	-	-	Attune® Acoustic Focusing Cytometer
Viability	LIVE/DEAD Fixable Red Dead Cell Stain	-	ThermoFisher Scientific	L23102	-	1:500	488	595	615	Life Technologies Corporation	20mW	2012	BL2	620 DLP // 574/26	Attune® Acoustic Focusing Cytometer
Viability	LIVE/DEAD Fixable Aqua Dead Cell Stain	-	ThermoFisher Scientific	L34957	-	1:500	405	375	512	Life Technologies Corporation	50mW	2012	VL2	575 DLP // 522/31	Attune® Acoustic Focusing Cytometer
DNA	Hoechst 33342	-	Sigma-Aldrich	14533	5mg/ml	1:500	405	340	510	Life Technologies Corporation	50mW	2012	VL1	410 DLP // 450/40	Attune® Acoustic Focusing Cytometer
**Isotype Controls and secondary detection**															
Mouse IgG2b	BV421	Mouse	Biolegend	400342	Lot specific	1:20	405	405	421	Life Technologies Corporation	50mW	2012	VL1	410 DLP //	Attune® Acoustic Focusing Cytometer
Mouse IgG1 κ	PE-Cy7	Mouse	BD Bioscience	557872	-	1:100	488	561	780	Life Technologies Corporation	20mW	2012	BL3	620 DLP //	Attune® Acoustic Focusing Cytometer
Streptavidin	PE	-	eBioscience	12-4317	0.2 mg/mL	1:2000	488	561	578	Life Technologies Corporation	20mW	2012	BL2	620 DLP // 574/26	Attune® Acoustic Focusing Cytometer

4.1. Compensation description	The automatic compensation for multicolor immunofluorescence analysis was performed using single color stained UltraComp eBeads™ Compensation Beads (Invitrogen, 01-2222-4). As a compensation controls for viability live or dead cells with diluted viability staining were used. For DNA stain live cells with/without DNA probe was used. Compensation matrix was calculated using FlowJo software.
4.2. Data transformation details	Not applicable
4.3.1. Gate description	Stained single cell suspensions were gated as follows: Nuclear cells – Hoechst 33342 (log) vs. FSC (lin); Single cells - FSC-H vs. FSC-A; Compact cell population (debris elimination) – FSC-A vs. SSC-A; Viable GFP+ cells – cells negative for LIVE/DEAD cell stain (LIVE/DEAD fluorescence (log) vs. GFP (log).<br>Specific fluorescence detection – according to appropriate isotype control or negative control. Detailed gating strategy for all figures is presented in Supplementary Figure 1.
4.3.2. Gate statistics	Percentage of positive cells in each gate was determined (% of cells from parental population, after gating for viable cells, single cells and compact cell population).
4.3.3. Gate boundaries	For detection of specific fluorescence of tested markers, gates were set according to appropriate isotype controls or negative controls in a manner that isotype control or negative control had less than 1% of positive cells.
